# Prevalence of porcine enterovirus 9 in pigs in Middle and Eastern China

**DOI:** 10.1186/1743-422X-10-99

**Published:** 2013-03-28

**Authors:** Shixing Yang, Yan Wang, Quan Shen, Wen Zhang, Xiuguo Hua

**Affiliations:** 1 School of Medical Science and Laboratory Medicine, Jiangsu University, 301 Xuefu Road, Zhenjiang, Jiangsu, 212013, PR China; 2 School of Agriculture and Biology, Shanghai JiaoTong University, 800 Dongchuan Road, Shanghai, 200240, PR China

**Keywords:** Porcine enterovirus 9, Pigs, China, Epidemiology, Phylogenetic analysis

## Abstract

Little information on the epidemiology and pathogenicity of porcine enterovirus 9 (PEV-9) is available. The present study investigated the prevalence of PEV-9 in pig populations in middle and eastern China using reverse transcriptase (RT)-PCR. All 14 sampled farms were positive for PEV-9 and the overall prevalence of infection in the studied pigs was 8.3% (37/447). There was a higher frequency of infection in pigs aged 10–15 weeks (12/119, 10.1%) than in pigs aged >20 weeks (5/103, 4.9%). A 313 nucleotide sequence from the 5^′^-UTR region of 37 Chinese PEV-9 positive samples had 96.1-100% sequence homology. On phylogenetic analysis, sequences clustered into two major groups, from which two representative strains were selected to determine the complete RNA-dependent RNA polymerase (RdRp) gene sequence. Phylogenetic analysis based on the RdRp gene suggested that PEV-9 strains from China formed a new subgroup. Piglets were inoculated orally with the PEV-9 strain identified in this study. Although most experimental pigs showed no clinical signs, almost all carried PEV-9 in one or more tissues after 6 days post-inoculation. The results of tissue histologic examination suggested that PEV9 can cause pathological changes in cerebrum and lung.

## Introduction

Porcine enteroviruses (PEV) belong to the family *Picornaviridae*[1-7]. Based on cytopathic effects (CPEs), replication properties in different host cell lines and serological assays, PEVs have been divided into three groups: CPE group I (serotypes 1–7 and 11–13), CPE group II (serotype 8) and CPE group III (serotypes 9 and 10) [5,8,9].

Although PEV infection is most frequently subclinical, some strains have been associated with a wide variety of clinical diseases. In Europe, two PEV-1 strains were isolated from an outbreak of polioencephalomyelitis [10,11] and were designated Teschen and Talfan strains. In addition, strains of various serotypes have been isolated from pigs showing polioencephalomyelitis [12-14] or no evidence of clinical disease [13]. This situation has obscured the relationship between virulence and serotypes/CPE types. So far, there are no reports showing that PEV-9 causes clinical disease in pigs.

During our studies to detect human enterovirus in water specimens using reverse transcriptase (RT)-PCR), we obtained unexpected sequences which showed high sequence homology to PEV-9 when a BLAST search (http://blast.ncbi.nlm.nih.gov/Blast.cgi) was performed against GenBank sequences. To investigate whether this virus is prevalent in the general pig population in China, we collected porcine faecal specimens from middle and eastern China and detected the PEV-9 genome using RT-PCR with specific primers. Pigs were infected experimentally to determine whether PEV-9 in the faecal specimens could infect specific pathogen free (SPF) pigs and cause clinical signs.

## Materials and methods

### Sampling

A total of 447 faecal samples were obtained from healthy pigs aged 4–26 weeks from March 2008 to May 2009. The porcine faecal samples were obtained from 14 pig farms in Anhui Province (middle China) and Shanghai City (eastern China), using sterile disposable gloves and swabs. We have got written permission from each farmer of the 14 pig farms. All the swine farms are formalized and have good sanitation status. All the samples were converted to 10% (W/V) suspensions in 0.01 M phosphate buffered saline (PBS) (pH 7.2- 7.4) immediately following the sampling. These samples were shipped, frozen, to our laboratory.

### Reverse transcriptase-PCR and nested PCR

**Figure 1:**
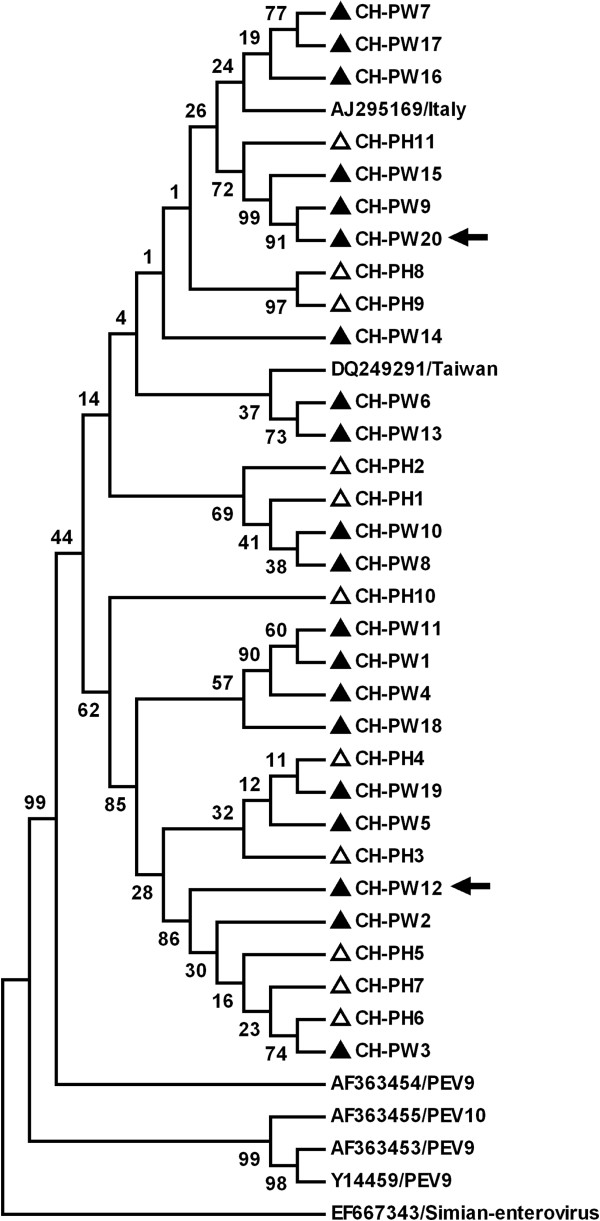
**Phylogenetic tree constructed by alignment of the 313 nt 5**^**′**^**-UTR sequence using Mega 4 software.** A simian enterovirus strain is included as an out-group. The isolates identified in this study are marked with black (Middle China) and white (Eastern China) triangles. The two arrows show the two representative strains which were selected to determine the sequence of the *RdRp* gene.

Faecal sample suspensions were clarified by centrifugation at 10,000 *g* for 10 min and 100 μL aliquots of the clarified material were used for viral RNA extraction. Total RNA was extracted using TRIzol (Invitrogen) and dissolved in 20 μL RNase-free water. The primers used for PEV-9 PCR have been described previously [15]: pev-9a forward primer 5^′^-GTACCTTTGTACGCCTGTTTTA-3^′^ and pev-9b reverse primer 5^′^-ACCCAAAGTAGTCGGTTCCGC-3^′^ for the first round of PCR and pev-9c forward primer 5^′^-CAAGCACTTCTGTTTCCCCGG-3^′^ and pev-9d reverse primer 5^′^-GTTAGGATTAGCCGCATTCA-3^′^ for the second round. This set of primers was designed to amplify a 313 nucleotide (nt) segment from the 5^′^-UTR, a highly conserved region of the genome, and were capable of detecting PEV-9 and PEV-10 (Figure 1).

RT- PCR was performed by using the TaKaRa RNA PCR kit with 4 μL RNA solution, 2 μL 25 mM MgCl_2_,1 μL 10x RT buffer, 1 μL 10 mM each deoxynucleotide, 20 pmol primer pev-9b, 10 U RNase inhibitor and 2.5 U avian myeloblastosis virus RT XL in a total volume of 10 μL. After incubation for 30 min at 42°C, the mixture was incubated for 5 min at 99°C to denature the products and then chilled on ice.

Five microlitres of the cDNA products were amplified by the universal RT-PCR assay using PerfectShot *Taq* (Loading dye mix) DNA polymerase (TaKaRa) in a total volume of 50 μL containing 10 μL cDNA, 25 uL loading dye mix and 25 pmol each of the sense and anti-sense primers. The PCR parameters for the first-round PCR included denaturation at 95°C for 5 min, followed by 35 cycles of denaturation for 50 s at 94°C, annealing for 55 s at 50°C, extension for 1 min at 72°C and a final incubation at 72°C for 5 min. Five microlitres of products of the first-round PCR were used as a template for the second-round PCR. The parameters for the second- round PCR were as for the first-round PCR.

### Nucleotide sequencing

To further elucidate the relationship between the virus strains in the present study and previously published porcine enterovirus strains, the *RdRp* gene sequence of two representative strains in this study were determined using primers designed according to the PEV-9 (GenBank NC_004441 and AF363453) and PEV-10 (AF363455) strains.

The PCR products were analysed in a 1.5% agarose gel containing 0.5 μg/mL ethidium bromide. The expected DNA band specific for PEV-9 was excised from the gel, purified with the AxyPrep DNA Gel Extraction kit (Axygen) and cloned into pMD T-vector (TaKaRa). Both strands of the inserted DNA amplicons were sequenced in an Applied Biosystems 3730 DNA Analyzer.

### Phylogenetic analysis

**Figure 2:**
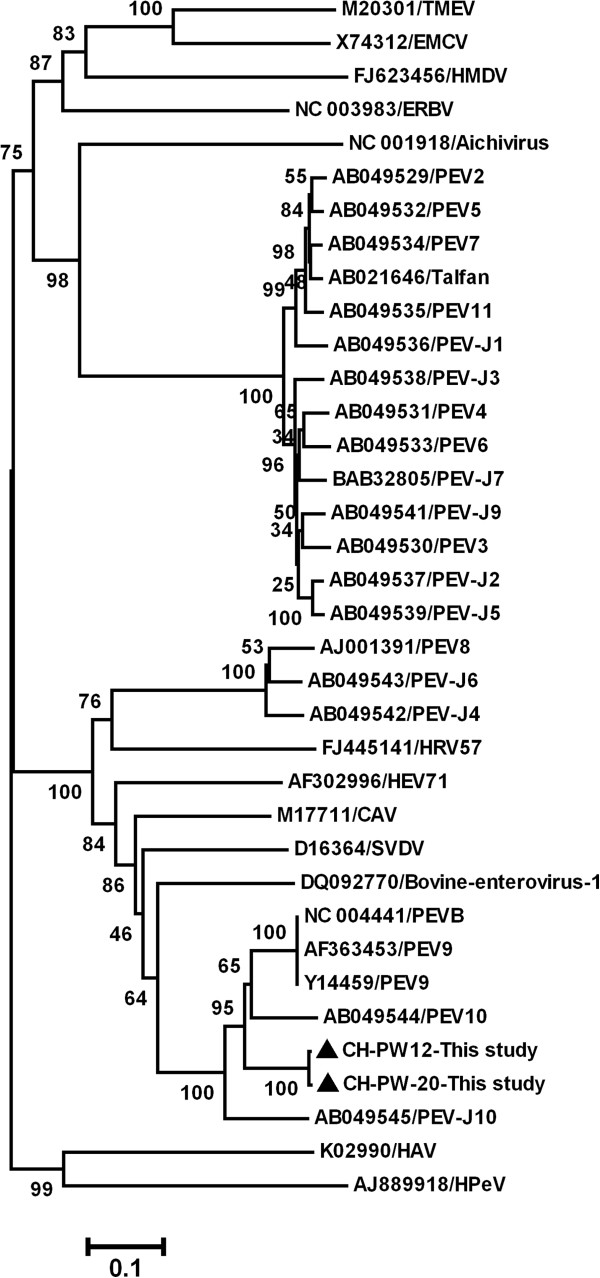
**Phylogenetic tree constructed by alignment of the complete *RdRp *gene sequence using Mega 4 software.** The isolates identified in this study are marked with black triangles.

**Figure 3:**
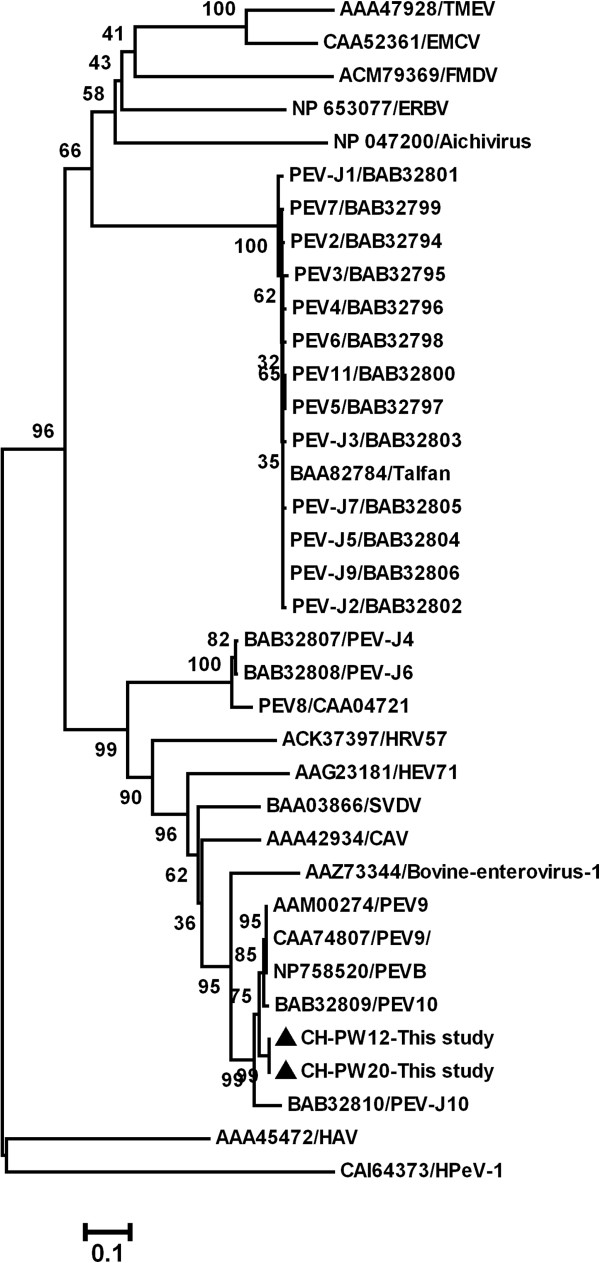
**Phylogenetic tree constructed by alignment of the RdRp amino-acid sequence using Mega 4 software.** The sequences determined in this study are marked with black triangles.

Nucleotide or predicted amino acid (aa) sequences were aligned using ClustaX v1.8 [16]. Phylogenetic analysis was performed using the Mega 4 software [17]. GenBank accession numbers of the previously published sequences used as references in this analysis are shown in Figures 1, 2 and 3. The sequences determined in current study were deposited in GenBank; isolate names are indicated in Figure 2 and the accession numbers are EF375319- EF375342.

### Experimental infection of pigs

To determine whether PEV-9 strains prevalent in pigs in China can infect pigs and cause clinical disease, one of the PEV-9 positive faecal samples was used for experimental inoculation. This sample was negative for other PEVs (including porcine teschoviruses, PEV1-8 and PEV-10), haemagglutinating encephalomyelitis virus, Aujeszky’s disease virus, porcine circovirus type 2, porcine reproductive and respiratory syndrome virus, classical swine fever virus, Japanese encephalitis virus, porcine transmissible gastroenteritis virus, porcine epidemic diarrhoea virus, porcine rotavirus, hepatitis E virus, porcine sapovirus, cytomegalovirus, porcine Torque-Teno virus and porcine parvovirus by RT-PCR/PCR methods. Supernatants were purified by passage through 0.22 μm microfilters (Millex-GV, Millipore) before virus inoculation or precipitation for electron microscopic observation.

Twelve 2-week-old Bama Mini-Pig SPF pigs (Veterinary Research Institute of SJTU), with an average weight of 500 g, were inoculated by the oral route using a feeding tube attached to a syringe with 3 mL virus suspension. Three other animals were inoculated with PBS as a control group. Each animal was kept in a separate cage. After inoculation, the pigs were observed until the end of the experiment at 21 days post-inoculation (PI). Two infected pigs were killed on each of days 2, 4, 6, 8, 14 and 21 PI. The three control animals were killed at 2, 8 and 14 days PI. At post-mortem examination, tissue samples were collected for virological examination, including samples of serum, faeces, liver, spleen, kidney, heart, lung, stomach, lymph node, cerebrum, cerebellum, brain stem, spinal cord and nasal cavity. Samples were frozen at −80°C. The study protocol was approved by Animal Care and Use Committee of Shanghai Research Center for Biomodel Organisms with a permit number of SRCBO62043034. We followed guidelines of the Shanghai Research Center for Biomodel Organisms during this study.

Liver, spleen, kidney, heart, lung, stomach, lymph node, cerebrum, cerebellum, brain stem, spinal cord and nasal cavity of the three pigs which exhibited pyrexia and one control were collected for histologic examination. Tissues were fixed in 10% neutral buffered formalin, routinely processed, sectioned at a thickness of 7 μm, and stained with hematoxylin and eosin.

## Results

### Occurrence of PEV-9

All 14 pig farms that were sampled were positive for PEV-9. The overall frequency of positive RT-PCR results was 37/447 (8.3%) and ranged from 2/29 (6.9%) to 6/47 (12.8%) on the 14 farms (Table 1). Pigs of 10–15 weeks of age had the highest frequency of positive RT-PCR results (12/119, 10.1%), while the pigs >20 weeks of age had the lowest frequency of positive RT-PCR results (5/103, 4.9%).

**Table 1:** Relationship between ages of pigs and frequency of PEV-9 by RT-PCR

**Age of pigs**	**Number of samples analysed**	**Number of positive samples (%)**
<10 weeks	120	11 (9.2)
10-15 weeks	119	12 (10.1)
16-20 weeks	105	9 (8.6)
>20 weeks	103	5 (4.9)
Total	447	37 (8.3)

### Sequence and phylogenetic analysis

Sequencing of the 313 nt 5^′^-UTR RT-PCR products of 37 PEV-9 positive samples demonstrated 96.1-100% sequence homology and identified 31 PEV-9 strains with distinct nt sequences. Phylogenetic analysis using the sequence alignments of these isolates and representative PEV-9 sequences in GenBank demonstrated two major groups; in group 1, two isolates from Taiwan (DQ249291) and Italy (AJ295169) clustered closely with 16 sequences obtained in the present study; in group 2, 15 isolates in the present study clustered together. These results suggested that there were two genetically distinct strains prevalent in pigs in middle and eastern China.

From the two major groups, two representative strains, CH-PW12 and CH-PW20 were selected and the complete RdRp gene sequences of these viruses were determined; the two complete RdRp nt sequences had 99.1% homology, whereas the predicted aa sequences were identical. Phylogenetic analysis was performed on the complete RdRp nt (Figure 2) and predicted aa sequences (Figure 3) using the two strains from the present study, 22 representative PEV strains available in GenBank and 12 other picornaviruses. In phylogenetic trees based on both nt and predicted aa sequences, PEVs were divided into three genetic groups. The two virus strains in the current study were classified into the cluster including PEV-9, PEV-10 and PEV-J10, but formed a new subgroup less related to PEV-9.

### Experimental animal infection

Three of 12 experimentally inoculated pigs exhibited pyrexia (39.5-40.5°C) at 2 days post-inoculation (dpi), which persisted for 2–6 days. Two of the three pigs with pyrexia exhibited flaccid paralysis of the hind limbs, which persisted to the time of necropsy. The other nine inoculated pigs, as well as the three control pigs, did not exhibit clinical signs.

Table 2 presents the distribution of PEV-9 antigen detected by RT-PCR in tissues of experimental pigs. PEV-9 RNA was first detected in the serum and spinal cord at 6 dpi. At 8 and 14 dpi, PEV-9 RNA was detected in serum, faeces, spleen, lung, brain stem and spinal cord of both or either of the two pigs examined in each group. At 21 dpi, there were fewer PEV-9 positive tissues than at 8 and 14 dpi. PEV-9 RNA was not detected in any tissues of pigs in the control groups.

**Table 2:** Distribution of PEV-9 detected by RT-PCR in tissues of experimental pigs (two pigs examined at each time point)

**Group**	**dpi**	**Clinical signs**	**Serum**	**Faeces**	**Liver**	**Spleen**	**Kidney**	**Heart**	**Lung**	**Stomach**	**Lymph node**	**Cerebrum**	**Cerebellum**	**Brain stem**	**Spinal cord**	**Nasal cavity**
1	2	+/−	−/−	−/−	−/−	−/−	−/−	−/−	−/−	−/−	−/−	−/−	−/−	−/−	−/−	−/−
2	4	−/−	−/−	−/−	−/−	−/−	−/−	−/−	−/−	−/−	−/−	−/−	−/−	−/−	−/−	−/−
3*	6	+/−	+/−	−/−	−/−	−/−	−/−	−/−	−/−	−/−	−/−	−/−	−/−	−/−	+/−	−/−
4	8	+/−	+/+	+/+	−/−	+/−	−/−	−/−	+/−	−/−	−/−	−/−	−/−	+/−	+/+	−/−
5	14	−/−	+/+	+/+	−/−	+/+	−/−	+/−	+/−	−/−	+/−	−/−	−/−	+/+	+/+	−/−
6	21	−/−	+/+	+/+	−/−	−/+	−/−	−/+	−/−	−/−	−/−	−/−	−/−	+/+	+/+	−/−

*dpi*, days post-inoculation; clinical signs are either present (+) or absent (−); for each entry in the table the two scores represents the presence (+) or absence (−) of viral gene detected by RT-PCR in the two pigs of each experimental group; * indicated the group in which the clinical sign of the pig is only pyrexia.

**Figure 4:**
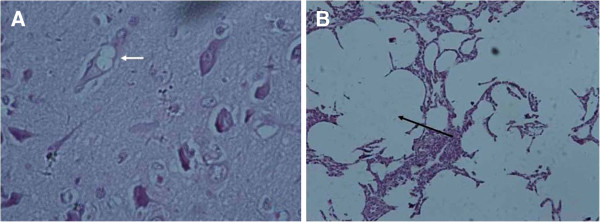
**Histopathologic changes observed in tissues of pigs.** (**A**), the neuron vacuolization (marked with arrow) in the cerebrum tissues of one pig which showed flaccid paralysis of the hind limbs; (**B**), the fracture of pulmonary bubbles (marked with arrow) in the pig which only showed pyrexia.

Figure 4A indicated the neuron vacuolization (marked with arrow) in the cerebrum tissues of one pig which showed flaccid paralysis of the hind limbs. Figure 4B showed the fracture of pulmonary bubbles (marked with arrow) in the pig which only showed pyrexia. There was no pathological change in the other tissues including those of the pig in control group.

## Discussion

Few systematic investigations of the prevalence of PEV-9 have been carried out in pigs in China or other parts of the world. In a recent study investigating the prevalence of enteroviruses in hot spring recreation areas of Taiwan, PEV-9 was detected in one hot spring downstream of a pig farm [18]. Our studies to detect calicivirus RNA in porcine faecal specimens often yielded unexpected sequences with high sequence homology to PEV-9. These findings implied that PEV-9 might be a common virus pathogen prevalent in pig groups in China and Taiwan. The aim of the present study was to perform a systematic survey of the prevalence of PEV-9 in pigs in middle and eastern China.

Our results suggest that PEV-9 is prevalent and widespread in the general pig population in middle and eastern China. Pigs >20 weeks of age had a lower frequency of PEV-9 by RT-PCR than pigs <15 weeks of age, which might be due to acquired immunity. A study of the seroprevalence of PEV-9 should be conducted to determine whether older pigs have a higher frequency of seropositivity for PEV9 than younger pigs.

Although some PEV strains have been associated with clinical disease, such as polioencephalomyelitis [10,11], enteric disease [13] and pneumonia [14], there are few reports showing that PEV-9 is pathogenic. In the present study, two-week-old SPF piglets were experimentally infected with a PEV-9 strain isolated from Chinese pigs. Two out of 12 pigs exhibited flaccid paralysis of the hind limbs; viral RNA could be detected in some tissues after 6 days of infection, especially the brain stem and spinal cord (Table 2). These results indicate that PEV-9 is pathogenic for pigs.

In the present study, the PCR primers for detection of PEV-9 were selected from the highly conserved 5^′^-UTR and were capable of detecting PEV-9 and PEV-10. However, only PEV-9 was detected in pigs in China. Phylogenetic analysis based on the 313 nt 5^′^-UTR segment divided Chinese strains of PEV-9 into two major groups. The complete RdRp gene of two representative strains were determined to further classify Chinese PEV-9 strains. The picornavirus RdRp is encoded in the 3D region at the 3^′^ end of the open reading frame and plays an important role in the virus life-cycle. Since RdRps are conserved among positive-strand RNA viruses, they have been analysed genetically in studies of picornavirus taxonomy [9,19,20].

On the basis of phylogenetic analysis of nt and predicted aa RdRp sequences, two Chinese PEV-9 formed a new subgroup related both to PEV-10 and PEV-9, which is inconsistent with phylogenetic analyses based on the 313 nt 5^′^-UTR sequence. The complete genomic sequence of the two representative virus strains in the current study should be determined to accurately clarify their relationships to the published PEV-9 and PEV-10.

The results of tissue histologic examination in the present study suggested that PEV9 can cause pathological changes in cerebrum and lung. The present systematic survey of the prevalence of PEV-9 in pigs in middle and eastern China will not only add to the weight of evidence on PEV epidemiology but also provide the diagnostic evidence for the clinical veterinarians.

## Conclusion

Taken together, the present study investigated the prevalence of PEV-9 in pig populations in middle and eastern China. All 14 sampled farms were positive for PEV-9 and pigs aged 10–15 weeks showed the highest positive rate of 10.1% (12/119). Sequence analysis based on the 5^′^-UTR region indicated that the Chinese PEV-9 isoaltes had 96.1-100% homology and clustered into two major groups. Phylogenetic analysis based on the RdRp gene suggested that PEV-9 strains in the present study formed a new subgroup. Experimental inoculation of 2-week-old pigs induced pyrexia (3/12) and flaccid paralysis (2/12), though the pathological changes in the central nervous system were not significant.

## Competing interests

The authors declare that they have no competing interests.

## Authors’ contributions

SY and XH conceived the study. SY, WZ, QS, and YW performed all the experiments. SY and WZ wrote the paper. All authors read and approved the final manuscript.
